# Effect of personal exposure to black carbon on changes in allergic asthma gene methylation measured 5 days later in urban children: importance of allergic sensitization

**DOI:** 10.1186/s13148-017-0361-3

**Published:** 2017-06-02

**Authors:** Kyung Hwa Jung, Stephanie Lovinsky-Desir, Beizhan Yan, David Torrone, Jennifer Lawrence, Jacqueline R. Jezioro, Matthew Perzanowski, Frederica P. Perera, Steven N. Chillrud, Rachel L. Miller

**Affiliations:** 1 Division of Pulmonary, Allergy, Critical Care Medicine, Department of Medicine, Columbia University College of Physicians and Surgeons, PH8E-101, 630 W. 168 St., New York, NY 10032 USA; 2 Division of Pediatric Pulmonary, Department of Pediatrics, College of Physicians and Surgeons, Columbia University, 630 W. 168 St., New York, NY 10032 USA; 3 Lamont-Doherty Earth Observatory, Columbia University, 61 Rt, 9 W Palisades, New York, 10964 USA; 4 Mailman School of Public Health, Department of Environmental Health Sciences, Columbia University, 722 W. 168 St., New York, NY 10032 USA; 5 Division of Pediatric Allergy, Immunology and Rheumatology, Department of Pediatrics, College of Physicians and Surgeons, Columbia University, PH8E-101, 630 W. 168 St., New York, NY 10032 USA

**Keywords:** Personal monitoring, Black carbon exposure, Changes in DNA methylation, Pediatric asthma, Allergic sensitization

## Abstract

**Background:**

Asthma gene DNA methylation may underlie the effects of air pollution on airway inflammation. However, the temporality and individual susceptibility to environmental epigenetic regulation of asthma has not been fully elucidated. Our objective was to determine the timeline of black carbon (BC) exposure, measured by personal sampling, on DNA methylation of allergic asthma genes 5 days later to capture usual weather variations and differences related to changes in behavior and activities. We also sought to determine how methylation may vary by seroatopy and cockroach sensitization and by elevated fractional exhaled nitric oxide (FeNO).

**Methods:**

Personal BC levels were measured during two 24-h periods over a 6-day sampling period in 163 New York City children (age 9–14 years), repeated 6 months later. During home visits, buccal cells were collected as noninvasive surrogates for lower airway epithelial cells and FeNO measured as an indicator of airway inflammation. CpG promoter loci of allergic asthma genes (e.g., interleukin 4 (IL4), interferon gamma (IFNγ), inducible nitric oxide synthase (NOS2A)), arginase 2 (ARG2)) were pyrosequenced at the start and end of each sampling period.

**Results:**

Higher levels of BC were associated with lower methylation of IL4 promoter CpG^−48^ 5 days later. The magnitude of association between BC exposure and demethylation of IL4 CpG^−48^ and NOS2A CpG^+5099^ measured 5 days later appeared to be greater among seroatopic children, especially those sensitized to cockroach allergens (RR [95% CI] 0.55 [0.37–0.82] and 0.67 [0.45–0.98] for IL4 CpG^−48^ and NOS2A CpG^+5099^, respectively), compared to non-sensitized children (RR [95% CI] 0.87 [0.65–1.17] and 0.95 [0.69–1.33] for IL4 CpG^−48^ and NOS2A CpG^+5099^, respectively); however, the difference was not statistically different. In multivariable linear regression models, lower DNA methylation of IL4 CpG^−48^ and NOS2A CpG^+5099^ were associated with increased FeNO.

**Conclusions:**

Our results suggest that exposure to BC may exert asthma proinflammatory gene demethylation 5 days later that in turn may link to airway inflammation. Our results further suggest that seroatopic children, especially those sensitized to cockroach allergens, may be more susceptible to the effect of acute BC exposure on epigenetic changes.

**Electronic supplementary material:**

The online version of this article (doi:10.1186/s13148-017-0361-3) contains supplementary material, which is available to authorized users.

## Background

Exposure to air pollution including black carbon (BC) or soot, a major component of fine particulate matter (PM)_2.5_, has been associated with worsening of asthma symptoms and airway inflammation among urban children [1, 2]. Understanding the temporality and mechanisms underlying the effects of BC exposure on airway inflammation may help direct environmental remediation, identify the time when health care needs are greater, and identify those at higher risk.

Cohort studies have suggested that acute (e.g., same day) and subacute (e.g., cumulative over days to one week) exposures to BC may exacerbate respiratory symptoms and increase airway inflammation among urban children [2–4]. Others have identified short-term lag effects (e.g., up to 5 days) of measures of air pollution on airway-related outcomes [2, 3, 5]. The individual dose of BC can vary significantly over days due to changes in personal behavior or activities [6] and over many days or 1 week related to variations in weather patterns in NYC [7–9]. Therefore, the wide variability in temporality between pollutant exposure and disease outcomes suggests that further refinement of the timeline of BC’s effects is needed. This can be achieved by understanding the timeline of the underlying mechanism that influences the relationship between BC and airway-related outcomes.

Environmental epigenetic regulation, including DNA methylation, is increasingly recognized as an important mechanism underlying the effects of air pollution on the development of complex diseases like asthma [10]. Exposure to BC, specifically, has been associated with altered DNA methylation of asthma genes in mouse studies [11], and in cohort studies comprised of elderly adults [12], and children [13]. Yet, these few studies have not pinpointed the timeline for environmental epigenetic regulation on asthma risk. Some studies suggest that epigenetic changes can occur relatively quickly and over days [14, 15] and in close proximity to short-term environmental exposures [16–18]. Other studies suggest that epigenetic patterns may be relatively stable or long-term [19, 20]. Moreover, the scarce research to date that has focused on air pollution-related epigenetic regulation of allergic asthma genes in a pediatric urban cohort [21] has not yet assessed the epigenetic consequence of exposure to BC and its possible effects measured days later. BC, and its diesel exhaust particle (DEP) sources, is important to immunoglobulin E (IgE) and allergic immune upregulation [22]. However, the differential susceptibility to BC-induced epigenetic changes by atopy, or by the urban asthma risk factor of cockroach sensitization [23, 24], has not been well studied.

Our objective was to determine the temporality of BC exposure on DNA methylation of genes and loci previously implicated in urban asthma and/or allergic sensitization (e.g., interleukin 4 (IL4), interferon gamma (IFNγ), inducible nitric oxide synthase (iNOS encoded by NOS2A), and arginase2 (ARG2)) [11, 12, 25–32] (Additional file 1: Figure S1) and how it may vary by seroatopy and cockroach sensitization. The key to defining this temporality is our novel determination of a child’s individual acute BC exposure level using personal monitoring. This approach integrates all fluctuating exposure levels by different time-activity patterns, which may also differ by age and sex and other key covariates [33]. We also repeatedly and noninvasively sampled the buccal mucosa to assess DNA methylation because this aerodigestive track epithelium is readily accessible in children [34], and altered methylation of other asthma/allergy genes here has been associated with airway inflammation in children [32]. Repeat measures of DNA methylation enabled us to control for previous DNA methylation levels, thus to assess changes in methylation over time. We measured DNA methylation using pyrosequencing to resolve small differences in methylation in response to acute BC exposure [35–37]. We also sought to determine the relationship between DNA methylation of allergic asthma genes and elevated fractional exhaled nitric oxide (FeNO), a key noninvasive measure of allergic airway disease [38, 39] that increases in response to air pollution exposure [1, 40]. FeNO has been linked specifically to buccal DNA methylation of NOS2A promoter and ARG2 [30, 32]. We hypothesized that daily personal exposure to BC would exert a short-term change in buccal cell methylation of allergic asthma genes 5 days later among urban children, and that the association between BC exposure and altered methylation may vary by seroatopy. We also hypothesized that DNA methylation of allergic asthma genes would affect FeNO levels.

## Methods

### Study population and personal air monitoring

Participants were recruited from the parent Columbia Center for Children’s Environmental Health (CCCEH) birth cohort [41]. For this nested study, 163 children who met criteria for age (9–14 years old) and underwent an evaluation for current asthma, determined by a specialized physician (allergist, pulmonologist) and a report of asthma symptoms or asthma medication use in the 12 months prior to enrollment [42], were recruited as previously described [43]. The study was approved by the Columbia University Institutional Review Board, and written informed consents and assents were obtained.

### Assessment of personal BC

**Fig. 1:**
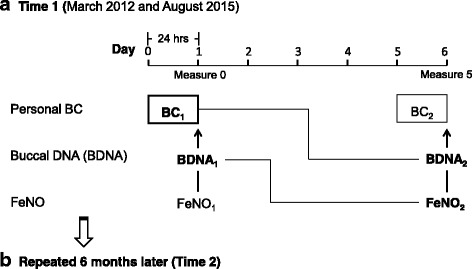
Study design: **a** time 1 (March 2012 and August 2015) and **b** repeated 6 months later (time 2). Personal BC was monitored for 24 h and collected on day 1 (BC_1_) and day 6 (BC_2_); Buccal DNA and FeNO were measured on d1 (BDNA_1_; FeNO_1_) and day 6 (BDNA_2_; FeNO_2_). Sampling was repeated 6 months later (time 2); measure five marks when effects of BC on methylation were measured 5 days later; *bolded* measures were used in current analyses. BC_2_ in a *thinner box* and FeNO_1_ were not used in the current analyses

Children wore a vest that contained a MicroAeth (Model AE51, Magee Scientific, Berkley, CA) personal BC monitor that was equipped with a nafion tube air inlet to avoid issues related to rapid changes in temperature and relative humidity in the breathing zone (i.e., vest collar). Compliance was assured by comparing accelerometer data from a device within the vest to the one worn on the child’s wrist, as described [43]. Personal BC levels were monitored every 5 min over two 24-h periods (BC_1_, BC_2_) 6 days apart between March 2012 and August 2015 (Fig. 1). Data were cleaned to remove false positive and negative data that can result from physical vibration following published methods [44, 45] and then averaged to yield a mean 24-h BC level. Over 90% of the first 24 h periods started on either Wednesday or Thursday to reduce variation in air pollution exposure by day of the week [46], and 60% were repeated 6 months later (*n* = 98, Additional file 1: Figure S2).

### Allergic sensitization

Total and cockroach allergen IgE levels were measured using Immunocap (Phadia, Uppsala, Sweden) [24]. Sera were collected at ages 7, 9, and 11, and data at age 9 or age 7 were used for the children that did not have a sample available at age 11. Because previous evidence suggested that combined exposure to traffic-related air pollution and cockroach allergen may worsen urban asthma morbidity [23, 24], primary analyses focused on German cockroach. Children were classified as seroatopic if total IgE ≥80 IU/mL and as cockroach sensitized if they had a specific cockroach IgE ≥0.35 IU/mL [24].

### Buccal sample collection and DNA extraction

Buccal DNA (BDNA) samples were collected on day 1 (BDNA_1_) and day 6 (BDNA_2_) using a CytoSoft cytology brush (Fischer Scientific, Pittsburgh, Pennsylvania) during home visits, following each 24-h BC collection (Fig. 1). DNA extractions were performed using the Gentra Puregene Buccal Cell kit (Qiagen, Germantown, Maryland). Homogeneity of the buccal cell population was assessed as described [47].

### DNA methylation

Targeted promoter region CpG loci were selected in regions of genes (i.e., IL4, IFNγ, NOS2A, ARG2) where methylation has been shown to be potentially responsive to traffic-related air pollution or has been implicated in allergy, asthma, and airway inflammation [11, 12, 25–32]. Loci with evidence of conservation between mouse and humans were especially targeted (Additional file 1: Figure S1). PCR and pyrosequencing were performed as reported [15, 47], using methylated and unmethylated DNA as positive and negative controls (Qiagen). BDNA_1_ and BDNA_2_ for each subject were amplified and pyrosequenced on the same 96-well plate. A duplicate buccal sample was collected in a subset and pyrosequenced as reported [47].

### Measurement of FeNO

FeNO was measured on day 1 (FeNO_1_) and day 6 (FeNO_2_) following each 24-h BC collection (Fig. 1), using the offline technique (GE Instruments, Boulder, CO). Three breath samples were collected in individual Mylar balloons at a flow rate of 83 ml/s [4]. Two ambient NO samples were simultaneously collected with FeNO using a nitric oxide analyzer (GE Instruments, Boulder, CO) to account for possible home environmental contamination of our sample. FeNO and ambient NO levels were averaged to obtain mean daily levels of FeNO and ambient NO, respectively. Fifteen percent of FeNO samples were collected but determined invalid due to ambient NO greater than 100 ppb.

### Statistical analyses

Analyses were restricted to children who had valid BC and methylation measures and available IgE data, with a final sample size of 143 (Additional file 1: Figure S2). BC and FeNO data were natural log-transformed to normalize skewed distributions. Descriptive statistics were analyzed using chi-square tests, Mann-Whitney tests, and Spearman correlations, as appropriate. Intraclass correlation coefficient (ICC) was calculated for correlations among multiple measures of personal BC (i.e., BC_1_ and BC_2_ at time 1) and FeNO (i.e., FeNO_1_ and FeNO_2_ at time 1). Due to the non-normal distribution of log-transformed methylation, the percent methylation of CpG sites within the IL4, IFNγ, and NOS2A genes were dichotomized at the upper tertile (high (1) vs low (0) methylation) [48]. As previously described [32], percent methylation of ARG2 was averaged across the three selected CpG sites then further dichotomized as “unmethylated (0)” vs “methylated (1)” if the average percent methylation was equal to zero or greater than zero. Methylation data were used as dichotomous variables (outcome variable) in analyses with personal BC and log-transformed continuous variables (predictor variable) in analyses with FeNO. Percent methylation values, instead of log-transformed data, were used for ARG2 in analyses with FeNO due to zero values in ARG2.

The associations between BC and changes in DNA methylation 5 days later were analyzed using a modified Poisson regression in generalized estimating equations (GEE) to estimate relative risks (RR) [49]. The results were expressed as RR of methylation in the highest tertile vs the second and lowest (as reference). The analyses were conducted using BC_1_ (on day 1) and BDNA_2_ (on day 6) with adjustment of BDNA_1_ (day 1) to assess delayed effects of BC on changes in DNA methylation over 5 days (Fig. 1). Models included covariates of race/ethnicity, sex, age, asthma diagnosis, heating season (October to April), obesity (body mass index (BMI) ≥age- and sex-specific 95th percentile of the year 2000 CDC growth charts), and seroatopy. The subjects were stratified by seroatopy or cockroach sensitization and adjusted models were run (but without controlling for seroatopy or cockroach sensitization) within each stratum. We further tested the interaction between BC and seroatopy on DNA methylation.

Multivariable linear regression models were used to examine the associations between DNA methylation and FeNO, after controlling for aforementioned covariates and ambient NO levels. The analyses were conducted using BDNA_1_ (on day 1) and FeNO_2_ (on day 6) to assess effects of DNA methylation on FeNO measured 5 days later (Fig. 1).

Secondary analyses include (1) analysis on the direct association between BC and FeNO measured 5 days later, (2) analysis on same-day association of DNA methylation with BC and FeNO, and (3) analysis on the association between DNA methylation and lung function outcomes.

Sensitivity analyses were conducted as follows: (1) reanalysis after removing one extreme personal BC concentration (exceeding 16 μg/m^3^), (2) reanalysis after controlling for chronic exposure, which was determined by the total percentage of secondary and connecting roads near a child’s home address at ages 7, 9, and 11 calculated using geographic information system from the CCCEH geospatial measures of the built and social environments database, (3) reanalysis after controlling for food intake by asking the question “In the past two hours, have you had anything to eat or drink?” and after eliminating current medication by asking the question “In the past 24 hours, have you taken any medications for asthma or allergies?,” and (4) reanalysis using the difference between BDNA_1_ and BDNA_2_ (i.e., BDNA_2_–BDNA_1_) as a continuous outcome in multivariable linear regression models. All analyses were performed using SPSS Statistic version 23.0 (SPSS Inc., Chicago, IL) where *p* < 0.05 was considered statistically significant. 

## Results

### Cohort characteristics

Characteristics of the 143 children along with personal BC and FeNO concentrations are presented in Table 1. There were no significant differences in demographic variables between seroatopic and non-atopic children except a higher frequency of obesity among the seroatopic children. Compared to the 584 CCCEH cohort children who did not meet the inclusion criteria for the nested study and thus were not enrolled, the enrolled children who were recruited to have a greater proportion of asthmatics also had a higher proportion of maternal asthma and cockroach sensitization (Additional file 1: Table S1).

**Table 1:** Cohort characteristics (*N* = 143)

Characteristic	Seroatopy^a^ (*n* = 75)	Non-atopy (*n* = 68)	*P* value^h^
Maternal ethnicity			0.34
Dominican	45/75 (60%)	46/68 (68%)	
African American	30/75 (40%)	22/68 (32%)	
Age mean [min–max], years	12.7 [10.4–14.3]	12.8 [10.5–14.0]	0.40
Girls	38/75 (51%)	39/68 (57%)	0.42
Maternal high-school degree or greater	40/74 (54%)	38/63 (60%)	0.46
Maternal asthma (+)	23/75 (31%)	19/68 (28%)	0.72
Prenatal ETS exposure^b^ (+)	23/74 (31%)	22/67 (33%)	0.62
Current ETS exposure^c^ (+)	7/67 (10%)	8/60 (13%)	0.82
Asthma^d^	44/75 (59%)	30/68 (44%)	0.08
BMI^e^ z score, median [IQR]	1.15 [1.43]	0.91 [1.35]	0.28
Overweight (≥85th percentile)	40/75 (53%)	33/68 (49%)	0.57
Obesity (≥95th percentile)	24/75 (32%)	12/68 (18%)	<0.05
Personal BC^f^ μg/m^3^, median [IQR]			
Day 1	1.23 [1.01]	1.27 [1.07]	0.68
Day 6	1.12 [1.25]	1.24 [1.29]	0.45
FeNO ppb^g^, median [IQR]			
Day 1	14.5 [15.3]	8.8 [8.85]	<0.01
Day 6	15.4 [12.4]	9.3 [8.81]	<0.01

*IQR* interquartile range

^a^Total IgE ≥80 IU/ML at age 7, 9, or 11 year

^b^Report of any smoker in the house during pregnancy

^c^Report of any smoker in the home during the 1-week sampling period

^d^Determined by a specialist physician using standardized criteria at age 5–12 years [42]

^e^Body mass index (BMI) calculated by weight (kg)/height (m)^2^, standard deviation (SD)

^f^Personal BC measured at time 1

^g^FeNO measured at time 1, *n* = 31 missing due to either invalid data or ambient NO >100 ppb

^h^Chi-square test for categorical variables and Mann-Whitney test for continuous variables (age, BMI z score, personal BC, and FeNO)

### Personal exposure to BC

Overall, urban children living in NYC were exposed to 1.21 μg/m^3^ of 24-h geometric mean levels of BC_1_. Personal BC levels did not differ by seroatopy (Table 1). Substantial changes in personal BC levels were observed within 5 days, with 28% of children experiencing greater than the interquartile range changes (IQR increase or decrease) between BC_1_ and BC_2_. Repeated measures of BC showed ICCs of 0.37 (5 days apart), indicating substantial within-subject variations in BC levels.

### Effects of BC on changes in DNA methylation of allergic asthma genes measured 5 days later

Descriptive statistics of DNA methylation on days 1 and 6 are shown in Additional file 1: Table S2. Further details also have been published [47]. DNA methylation of allergic asthma genes measured on day 1 (BDNA_1_) correlated weakly with those measured on day 6 (BDNA_2_) (Additional file 1: Figure S3). The methylation levels at each of the CpG loci on day 1 (BDNA_1_) did not differ by seroatopy (Mann-Whitney test, *p* > 0.05). Higher levels of BC were associated with lower methylation of IL4 promoter CpG^−48^ 5 days later, with and without controlling for covariates, including previous day 1 methylation of IL4 CpG^−48^ (Table 2). Similar associations were not apparent between BC and DNA methylation of other asthma gene promoters (i.e., IFNγ, NOS2A, and ARG2) in unadjusted and adjusted models (Table 2, *p* > 0.05).

**Table 2:** Effects of personal BC exposure on DNA promoter region methylation measured 5 days later: RR of methylation in the highest tertile vs the second and lowest (as reference)

		Overall effects, RR [95% CI]	
Gene	CpG promoter region^a^	Unadjusted	Adjusted^b^
IL4	−326	0.82 [0.63–1.06]	0.82 [0.63–1.07]
	−48	0.75 [0.58–0.96]^*^	0.77 [0.61–0.97]^*^
IFNγ	−186	1.15 [0.86–1.53]	1.18 [0.88–1.58]
	−54	0.91 [0.68–1.20]	0.92 [0.68–1.26]
NOS2A	+5099	0.84 [0.64–1.09]	0.82 [0.65–1.03]
	+5106	1.20 [0.91–1.59]	1.21 [0.94–1.55]
ARG2	−32, −30, and −26^c^	1.06 [0.95–1.18]	1.04 [0.94–1.15]

Note: 86 of the 143 children underwent repeat testing 6 months later allowing for *n* = 229 total observations analyzed

^*^ *p* value <0.05

^a^CpG position relative to the transcriptional start site

^b^Adjusted for race/ethnicity, sex, age, asthma diagnosis, obesity, seroatopy, heating season, and DNA methylation at day 1

^c^Average methylation of ARG2 CpG sites of −32, −30, and −26

**Fig. 2:**
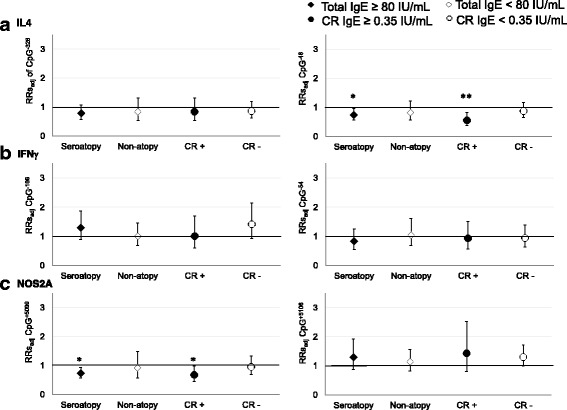
Effects of personal BC exposure on DNA promoter region methylation 5 days later of **a** IL4 (CpG^−326^, CpG^−48^, **b** IFNγ (CpG^−186^, CpG^−54^), and **c** NOS2A (CpG^+5099^, CpG^+5106^), stratified by allergic sensitization. Relative risk (RR) estimates of DNA methylation of asthma genes and 95% confidence interval (CI), for a unit increase in log BC concentrations among seroatopic (♦ *N* = 75), non-atopic (⋄ *N* = 68), cockroach-sensitized (• CR+ *N* = 55), and non-sensitized (○ CR− *N* = 88) children, adjusting for race/ethnicity, sex, age, asthma diagnosis, obesity, season, and DNA methylation on day 1. *p < 0.05 and **p < 0.01

When stratified by seroatopy, the magnitude of association between BC exposure and demethylation of IL4 CpG^−48^ and NOS2A CpG^+5099^ 5 days later appeared to be greater among seroatopy, compared to non-atopy (Fig. 2 and Additional file 1: Table S3). A similar pattern was observed when stratified by cockroach sensitization with smaller RR’s (Fig. 2 and Additional file 1: Table S3). However, a significant interaction between BC and seroatopic or cockroach sensitization on DNA methylation of asthma genes was not observed (Additional file 1: Table S4. *P*_interaction_ > 0.05).

### Effects of DNA methylation on FeNO levels measured 5 days later

Median FeNO was 12.3 ppb (min–max = 2.54–85.3 ppb) on day 6 (FeNO_2_). Seroatopic or cockroach-sensitized children exhibited significantly higher levels of FeNO compared to non-atopic children (e.g., median 15.4 vs 9.3 ppb for seroatopic vs non-atopic children; Mann-Whitney test; *p* < 0.001). ICCs of repeated measures of FeNO were 0.81 (5 days apart).

**Fig. 3:**
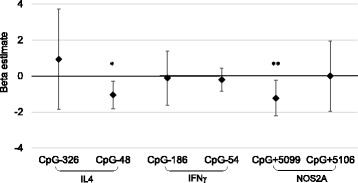
Effects of DNA methylation on FeNO measured 5 days later. Beta coefficient of FeNO and 95% CI for a unit increase in log percent DNA methylation presented. Model adjusted for race/ethnicity, sex, age, asthma diagnosis, obesity, season, and ambient NO on day 6. Sixteen children were removed from the analyses of FeNO and DNA methylation due to either high ambient NO levels (>100 ppb) or equipment failure, resulting in a sample size of 127. *p < 0.05 and **p < 0.01

In multivariable linear regression models, lower DNA methylation of IL4 CpG^−48^ and NOS2A CpG^+5099^ were associated with higher FeNO, with adjustment of seroatopy and ambient NO, 5 days later (Fig. 3 and Additional file 1: Table S5). However, the associations of FeNO with DNA methylation of other asthma gene promoters (i.e., IFNγ and ARG2) were not significant in adjusted models (Additional file 1: Table S5, *p* > 0.05).

### Secondary analyses

We did not observe a direct association between BC exposure and FeNO measured 5 days later in an adjusted model (*p* > 0.05). We explored same-day associations between BC and DNA methylation, using both day 1 (BC_1_ and BDNA_1_) and day 6 (BC_2_ and BDNA_2_) measures in adjusted models. An overall association between BC exposure and DNA methylation of IL4 CpG^−48^ and NOS2A CpG^+5099^ measured on the same day was not significant (*p* > 0.05). Similarly, when same-day associations between DNA methylations and FeNO were examined, previously observed significant associations of FeNO with IL4 CpG^−48^ and NOS2A CpG^+5099^ were not detected (*p* > 0.05).

Second, we explored the associations between IL4 and NOS2A methylation and FeNO by cockroach sensitization. The effects of IL4 CpG^−48^ and NOS2A CpG^+5099^ on FeNO measured 5 days later also seemed more apparent among cockroach-sensitized children, compared to non-sensitized children (beta estimate (*p* value) = −0.91 (0.03) and −1.31 (0.01) for IL4 CpG^−48^ and NOS2A CpG^+5099^, respectively). To verify the possible clinical impact of these associations, we repeated the analyses on outcomes related to lung function (methods in Additional file 1). In multivariable linear regression models, demethylation of NOS2A CpG^+5099^, but not IL4 CpG^−48^, was associated with decreased forced expiratory volume in 1 s/forced vital capacity, (FEV_1_/FVC), forced expiratory flow at 25–75% of forced vital capacity (FEF_25–75_), peak expiratory flow rate (PEFR), 5 days later (beta estimate (*p* value) = 0.16 (<0.001), 0.56 (<0.001), and −1.31 (<0.01) for FEV_1_/FVC, FEF_25–75_, and PEFR, respectively).

### Sensitivity analyses

First, after removing one extreme data point (BC ≥16 μg/m^3^), the main findings in Table 2 and Fig. 2 remained (data not shown). Second, when the indicator of chronic exposure (i.e., the total percentage of secondary and connecting roads near a child’s home address) was further controlled in adjusted model, the effects of BC on IL4 CpG^−48^ measured 5 days later was replicated (RR [95% CI] 0.76 [0.61–0.95]; *p* = 0.017). Further, in models stratified by seroatopy, the main findings in Fig. 2 remained similar after controlling for chronic exposure (RR [95% CI] 0.72 [0.55–0.94]; *p* = 0.014 and 0.73 [0.57–0.94]; *p* = 0.015 for IL4 CpG^−48^ and NOS2A CpG^+5099^, respectively). A consistent pattern also was observed when stratified by cockroach sensitization (RR [95% CI] 0.55 [0.37–0.83]; *p* = 0.004 and 0.67 [0.45–0.98]; *p* = 0.038 for IL4 CpG^−48^ and NOS2A CpG^+5099^, respectively). When we controlled for food intake and eliminated those on asthma/allergy medications, the main findings in Table 2 persisted (data not shown). Last, when the difference between BDNA_1_ and BDNA_2_, instead of dichotomous outcome, was used in the main analysis, a similar trend was observed for IL4 CpG^−48^ (but not for other loci). The higher levels of BC were associated with a decrease in methylation of IL4 CpG^−48^ over 5 days (beta estimate (*p* value) = −1.75 (0.048)) in multivariable linear regression model (Additional file 1: Table S6).

## Discussion

In a cohort of urban children, we found associations of 24-h averaged BC measured by personal monitoring on DNA hypomethylation at the IL4 promoter measured 5 days later, even after controlling for the previous methylation levels. The effects on lowering IL4 and NOS2A methylation 5 days later appeared to be greater among the seroatopic and cockroach-sensitized children. We also found that IL4 and NOS2A demethylation was associated with elevated FeNO 5 days later. Together, our results suggest that individual acute exposure to BC may exert delayed allergic asthma proinflammatory gene demethylation effects that in turn may link to airway inflammation. The potential susceptibility among those sensitized to cockroach observed here also may help explain previous links between cockroach sensitization and inner city asthma in children [50].

One of the strengths of this study is the assessment of BC using a real-time personal monitor. This approach can capture the spatial and temporal variability in traffic-related air pollution in urban settings [51, 52] and consider more accurately the individual mobility of older children traveling over the course of a day between school, home, and other urban locations. Presumably, measures of personal BC avoid misclassification of measures of time spent in specific locations that may supplement estimates from land use regression (LUR) modeling [53]. Further, the low ICC value of personal BC over 5 days substantiates these fluctuations in individual exposure. Another strength was the repeat buccal sample collection intended to discern some of the dynamic changes of epigenetic regulation in response to environmental exposure. We also utilized a well-phenotyped prospective birth cohort study with a rich database on each child’s clinical information and chronic exposure measurements.

Our findings of an effect of short-term (24-h) BC on changes in DNA methylation and of methylation on FeNO, each 5 days later, among urban children are novel. In comparison, we did not detect the same-day effect of BC on lower methylation of IL4 CpG^−48^ and NOS2A CpG^+5099^. This holds true when chronic exposure to traffic-related air pollution was further controlled in the analysis, suggesting that recent acute exposure to BC may play an important role in the dynamics of epigenetic regulation. Several controlled human studies have reported short-term DNA methylation changes induced by particles (i.e., concentrated ambient particles or diesel exhaust) [35, 54]. One study with older adults demonstrated that IQR increases in BC measured during the preceding 4 weeks were associated with significant decreases in IFNγ methylation among never smokers [55]. Otherwise, to date, studies on the association between BC and other traffic-related air pollutants and DNA methylation are scarce, especially among children. But in one pediatric cohort, 7-day averaged levels of fine particulate matter were associated with demethylation of NOS2A [30, 56]. Our study provides the first evidence that short-term BC exposure may induce a delayed effect on changes of IL4 and NOS2A demethylation among children.

We found seemingly delayed effects of DNA hypomethylation of two allergic asthma genes on FeNO levels measured 5 days later. Lower methylation, consistent with the potential for greater gene expression, could underlie the reported association between measures of air pollution and increases in FeNO over this time course [1, 4, 40, 57]. Analytically, here, we did not find that methylation mediated an association between BC and FeNO due to a lack of direct association between BC exposure and FeNO measured 5 days later. These findings may be a result of the study timeline, as we assessed in parallel a 5-day period of BC on methylation and a 5-day period of methylation on FeNO (Fig. 1). While the primary objective was to assess the association of BC with allergic asthma gene methylation 5 days later, our results may indicate there was a 10-day effect between BC exposure and FeNO levels. Given the high correlation between FeNO measurements over 5 days, this signal may be small. Although, our secondary analyses revealed that there was no same-day association between methylation and FeNO. Alternately, additional unmeasured environmental exposures, despite our numerous controls, could be inducing the epigenetic alterations associated with airway inflammation, or additional CpG loci or genes could be inducing epigenetic regulation of FeNO; although, insufficient power to a detect mediation effect could have been a limitation. We also acknowledge that the absence of statistical evidence of mediation by methylation on FeNO does raise some uncertainty as to underlying mechanisms. In improving the biological plausibility, we replicated the previously reported association between NOS2A methylation and lung function found in elderly men [58] and among urban children and according to a specific acute timeline.

Interestingly, we observed that the associations of DNA methylations with BC and FeNO appeared stronger among seroatopic and cockroach-sensitized children, compared to non-atopic children. Several pediatric cohorts, including ours, have investigated the effects of air pollution by asthma phenotypes [1, 59, 60], but not in the context of environmental epigenetic regulation. One study reported stronger associations between polycyclic aromatic hydrocarbon (PAH) exposure and forkhead box P3 (FOXP3) methylation among nonasthmatic rhinitis children, compared to those among non-atopic or asthmatic children with/without rhinitis [21]. We previously reported that the association between PAH exposure and asthma was more prominent among non-atopic children, compared to that among seroatopic children [59]. However, these studies differed by age (10–14 vs 5–6 years) and air pollutants (BC vs PAH). Here, we presented that seroatopic children, especially those sensitized to cockroach allergens, may be more susceptible to the effects of BC on epigenetic changes and possibly airway inflammation, as a possible mechanism underlying BC’s reported adjuvant effects on allergy [22].

We focused on epigenetic changes among known allergic asthma genes and previously reported regions of altered epigenetic regulation. By doing so, we were able to replicate the importance of the IL4 CpG^−48^, previously shown and validated as associated with urban asthma [25]. We extended this finding to show its susceptibility specifically to acute individual exposure to BC. We also replicated the importance of NOS2A, previously shown to be associated with airway inflammation among children in Southern California/LA area [30, 56]. While both studies found the link between traffic-related air pollution and demethylation of NOS2A CpG^+5099^ in buccal cells, new elements in our study design included the personal exposure assessment with specific focus on BC, capture of effects of an even shorter duration of exposure (24-h average instead of 7-day cumulative average), and a delayed effect of exposure. An additional distinction was the consideration of cockroach sensitization.

The reductions in allergic asthma gene methylation measured here as relative risks captured the relatively small yet significant changes in levels of methylation that were predicted to occur in daily urban living and following the behaviors of older children. We acknowledge that these small differences in single CpG sites, which essentially indicate changes among a low percent of cells within the tissues, may only partially explain the underlying immunopathogenesis. But in specific contexts, they may be reasonable surrogate measures or indicate functional differences at the cellular level [55]. Small differences (1–3%) in methylation have been shown in previous studies to associate with several fold changes in gene expression [36, 61]. In addition, small mean differences in asthma candidate gene methylation that ranged less than 1% were associated with prenatal exposure to farm or asthma [62] and are common in environmental epigenetic studies [37]. Similar small (approximating 0.1) increases in CpG methylation β values were associated with a 4.7% increase in body mass index and an 11.8 increase in fat mass index (FMI) [63]. We also related these small differences in methylation to statistically significant differences in FeNO, supporting their clinical relevance. The paradigm that the small magnitude of effect sizes found here carries additional weight because we essentially replicated previous gene links but with much finer resolution of the exposure, its timing, and relative percent methylation by CpG site. The findings also support previous evidence [34, 35, 64], although mixed [65], that associations can vary across gene areas.

We acknowledge several additional limitations, including the relatively small sample size necessitated by our relatively comprehensive and repeated exposure assessment. The IL4 CpG^−326^ findings are in the same direction as those of CpG^−48^ in the analysis with BC, especially in stratified analysis by seroatopic, although not statistically significant. One explanation may be that we had insufficient statistical power to detect a difference at CpG^−326^ related to personal BC exposure. The small differences in methylation by BC exposure may be difficult to differentiate small differences in methylation from noise particularly at the lowest end of the spectrum as measured for ARG2 or from those measured in duplicate data [47]. We did not perform technical replicates because this procedure is not recommended by the manufacturer (Qiagen) due to the presence of several internal controls; however, blinded duplicates at extremes of methylation (e.g., very low or high) may be poorly correlated. Further, the use of categorized DNA methylation data (e.g., the highest tertile for IL4, IFNγ, and NOS2A and any methylation greater than 0 for ARG) could miss some information from that of continuous measures. Nonetheless, most of our findings remained robust during sensitivity analyses allowing us to draw some important conclusions. We targeted specific CpG loci in the promoter region, which was previously implicated in air pollution-related asthma [11, 12, 25–32] (Additional file 1: Figure S1); however, important epigenetic control mechanisms in the gene body beyond the promoter could be missed [66]. We do not anticipate confounding by nonhuman DNA in our buccal collections as mouths were rinsed prior to cell collections. Indeed, the main results remain the same, even after considering food intake and medication use. While unmeasured confounding is always a possibility in cohort research, the repeat measure design was intended to minimize its contribution. Even though we did detect the stronger association between BC and methylation among seroatopic and cockroach-sensitized children compared to non-atopic children, our analyses of effect modification indicated that they are not statistically different. Finally, sensitization to different specific allergens, such as tree, grass, ragweed, or mold, also may contribute to epigenetic changes in response to BC exposure.

## Conclusions

These findings offer new evidence of the relatively acute and delayed temporal effects of environmental epigenetic regulation of allergic asthma genes and their differential susceptibility by allergic phenotype. Further findings suggest associations between environmental epigenetic regulation and airway inflammation. Together, these findings inform on the dynamics of environmental epigenetic regulation among a highly susceptible, mobile and difficult to capture pediatric age group.

## Additional file

Additional file 1: Figure S1. Conserved promoter regions. Black lines mark loci that are conserved between human and mouse in the promoter region of IL4, IFNγ, and ARG2. White areas are not conserved. Conserved regions were identified using Standard Nucleotide BLAST (blastn for more dissimilar regions; https://blast.ncbi.nlm.nih.gov/Blast.cgi.) for the 400 nucleotides upstream of the transcriptional start site (TSS) in the human sequence. The NOS2A promoter region under investigation is not conserved between mice and human. **Figure S2**: Schematic demonstration of collected measures. Numbers in the box represent the number of participants. *N*:*n* = number of repeat subjects: number of observations. Grey dotted box indicates two measures (both time 1 and time 2, 6 months apart) available and white box only one measure (Time 1) available. *N* = 10 participants dropped due to invalid personal or residential air pollution measures. *N* = 17 participants were further excluded from the analysis due to missing total IgE (*N* = 16) and invalid DNA methylation due to technical failures in the laboratory (*N* = 1), resulting in *N* = 136 of the final sample size. **Figure S3**: Correlations between day 1 and day 6 buccal cell DNA methylations of (a) IL4 (CpG^−326^,CpG^−48^, (b) IFNγ (CpG^−186^,CpG^−54^), and (c) NOS2A (CpG^+5099^, CpG^+5106^) and (d) ARG2 (average methylation of CpG^−32^, CpG^−30^, and CpG^−26^), Spearman correlation coefficient presented. (DOCX 466 kb)

## Abbreviations

ARG2: Arginase 2
BC: Black carbon
BDNA: Buccal DNA
CCCEH: Columbia Center for Children’s Environmental Health
DEP: Diesel exhaust particle
EC: Elemental carbon
FeNO: Fractional exhaled nitric oxide
FMI: Fat mass index
FOXP3: Forkhead box P3
GEE: Generalized estimating equations
ICC: Intraclass correlation coefficient
IFNγ: Interferon gamma
IgE: Immunoglobulin E
IL4: Interleukin 4
iNOS: Inducible nitric oxide synthase
IQR: Interquartile range
LUR: Land use regression
NYC: New York City
PM: Particulate Matter
RR: Relative Risk
