# Supplementary material for: Effect of personal exposure to black carbon on changes in allergic asthma gene methylation measured 5 days later in urban children: importance of allergic sensitization
Source: Clin Epigenetics. 2017 Jun 2;9:61. doi: 10.1186/s13148-017-0361-3 (PMC5457544; doi:10.1186/s13148-017-0361-3)
Supplement: Additional file 1: Figure S1. — Conserved promoter regions. Black lines mark loci that are conserved between human and mouse in the promoter region of IL4, IFNγ, and ARG2. White areas are not conserved. Conserved regions were identified using Standard Nucleotide BLAST (blastn for more dissimilar regions; https://blast.ncbi.nlm.nih.gov/Blast.cgi.) for the 400 nucleotides upstream of the transcriptional start site (TSS) in the human sequence. The NOS2A promoter region under investigation is not conserved between mice and human. Figure S2: Schematic demonstration of collected measures. Numbers in the box represent the number of participants. N:n = number of repeat subjects: number of observations. Grey dotted box indicates two measures (both time 1 and time 2, 6 months apart) available and white box only one measure (Time 1) available. N = 10 participants dropped due to invalid personal or residential air pollution measures. N = 17 participants were further excluded from the analysis due to missing total IgE (N = 16) and invalid DNA methylation due to technical failures in the laboratory (N = 1), resulting in N = 136 of the final sample size. Figure S3: Correlations between day 1 and day 6 buccal cell DNA methylations of (a) IL4 (CpG−326,CpG−48, (b) IFNγ (CpG−186,CpG−54), and (c) NOS2A (CpG+5099, CpG+5106) and (d) ARG2 (average methylation of CpG−32, CpG−30, and CpG−26), Spearman correlation coefficient presented. (DOCX 466 kb) [file 13148_2017_361_MOESM1_ESM.docx]

**Table S1.** Cohort characteristics

| Characteristic | Participants included^a^ (n=143) | Not included  (n=584) | P-value^e^ |
| --- | --- | --- | --- |
| Maternal ethnicity |  |  | 0.69 |
| Dominican | 91/143 (64%) | 382/584 (65%) |  |
| African American | 52/143 (36%) | 202/584 (35%) |  |
| Age mean [min-max], yrs | 12.8 [10.4-14.3] | - |  |
| Girls | 77/143 (54%) | 299/584 (51%) | 0.57 |
| Maternal high school degree or greater | 78/137 (57%) | 378/576 (66%) | 0.06 |
| Maternal asthma (+) | 42/143 (29%) | 121/584 (21%) | **0.03** |
| Prenatal ETS exposure^b^ (+) | 45/141 (32%) | 201/576 (35%) | 0.50 |
| Current ETS exposure^c^ (+) | 15/127 (12%) |  |  |
| Asthma^d^ | 74/143 (52%) | 96/366 (26%) | **<0.001** |
| Total IgE (≥ 80 IU/mL) | 75/143 (52%) | 112/260 (43%) | 0.07 |
| Cockroach sensitization (≥ 0.35 IU/mL) | 55/143 (38%) | 68/273 (25%) | **0.004** |

^a^Includes children in nested study with complete data available for current analysis. Participants excluded if personal and residential indoor sampling data not collected (n=564), invalid air pollution measures (n=10), missing total IgE (n=16), or invalid pyrosequencing measures (n=1)

^b^Report of any smoker in the house during pregnancy.

^c^Report of any smoker during 1-week sampling period.

^d^Asthma was determined by a specialist physician using standardized criteria at age 5-12 year [1]

^e^P-value calculated using Mann-Whitney test of Chi-tests

**Table S2.** Descriptive statistics of DNA methylation on Day 1 and 6

|  |  | Day 1 | | Day 6 | |
| --- | --- | --- | --- | --- | --- |
| Gene | CpG promoter region^a^ | Mean (SD) | 95% CI for mean [lower, upper] | Mean (SD) | 95% CI for mean [lower, upper] |
| IL4 | -326 | 93.1 (2.8) | [92.6, 93.5] | 93.0 (2.1) | [92.7, 93.4] |
|  | -48 | 87.5 (7.7) | [86.2, 88.7] | 87.2 (5.8) | [86.3, 88.2] |
| IFNγ | -186 | 73.6 (5.2) | [72.8, 74.5] | 73.7 (6.1) | [72.7, 74.7] |
|  | -54 | 80.2 (7.2) | [79.0, 81.4] | 80.4 (4.5) | [79.7, 81.1] |
| NOS2A | +5099 | 59.6 (5.8) | [58.6, 60.5] | 60.7 (4.8) | [59.9, 61.5] |
|  | +5106 | 82.0 (4.7) | [81.2, 82.8] | 82.1 (4.5) | [81.4, 82.9] |
| ARG2 | -32, -30, and -26^c^ | 0.99 (0.73) | [0.87, 1.1] | 0.92 (0.81) | [0.79, 1.0] |

SD (standard deviation); CI (Confidence interval); n=143

**Table S3.** Effects of personal BC exposure on DNA promoter region methylation measured 5 days later: Stratified by allergic sensitization: RR of methylation in the highest tertile vs the second and lowest (as reference)

|  | CpG | RR_adj_^a^ [95% CI] | | | | |
| --- | --- | --- | --- | --- | --- | --- |
| Gene | Promoter region^b^ | Seroatopy^c^ | Non-atopy |  | Cockroach sensitization^d^ | Non-sensitization^e^ |
| IL4 | -326 | 0.79 [0.58-1.08] | 0.84 [0.54-1.32] |  | 0.84 [0.54-1.31] | 0.86 [0.62-1.20] |
|  | -48 | **0.74 [0.56-0.97]*** | 0.82 [0.56-1.22] |  | **0.55 [0.37-0.82]**** | 0.87 [0.65-1.17] |
| IFNγ | -186 | 1.29 [0.89-1.86] | 1.00 [0.68-1.46] |  | 1.01 [0.60-1.69] | 1.41 [0.93-2.15] |
|  | -54 | 0.83 [0.55-1.25] | 1.04 [0.68-1.61] |  | 0.93 [0.57-1.50] | 0.94 [0.64-1.39] |
| NOS2A | +5099 | **0.73 [0.57-0.93]*** | 0.92 [0.57-1.48] |  | **0.67 [0.45-0.98]*** | 0.95 [0.69-1.33] |
|  | +5106 | 1.29 [0.87-1.92] | 1.14 [0.82-1.57] |  | 1.43 [0.81-2.53] | 1.30 [0.99-1.71] |
| ARG2 | -32, -30, and -26^f^ | 1.07 [0.93-1.22] | 0.99 [0.84-1.16] |  | 1.05 [0.90-1.22] | 1.04 [0.89-1.23] |

Note: N=Number of subjects and n: number of observations including repeat measurements 6 months later; [N:n]=[75:122], [68:107], [55:89], and [88:140] for seroatopic, nonatopic, cockroach sensitized, and non-sensitized children, respectively.

86 of the 143 children underwent repeat testing 6 months later allowing for n=229 total observations analyzed.

^a^Model adjusted for race/ethnicity, sex, age, asthma diagnosis, obesity, heating season, and DNA methylation on Day 1

^b^CpG position relative to the transcriptional start site

^c^Positive total IgE ≥ 80 IU/mL

^d^Cockroach IgE ≥ 0.35 IU/mL

^e^Cockroach IgE < 0.35 IU/mL

^f^Average methylation of ARG2 CpG sites of -32, -30, and -26; *p-value <0.05, and **p<0.01

**Table S4.** P-value for interactions between personal BC and seroatopy and cockroach sensitized on DNA methylation measured 5 days later:

|  |  | P-value for interaction^a^ on DNA methylation | |
| --- | --- | --- | --- |
| Gene | CpG Sites^b^ | BC × Seroatopy^c^ | BC × Cockroach sensitization^d^ |
| IL4 | -326 | 0.83 | 0.83 |
|  | -48 | 0.60 | 0.14 |
| IFNγ | -186 | 0.19 | 0.57 |
|  | -54 | 0.45 | 0.72 |
| NOS2A | +5099 | 0.46 | 0.29 |
|  | +5106 | 0.63 | 0.85 |
| ARG2 | -32, -30, and -26^e^ | 0.41 | 0.94 |

Note: 86 of the 143 children underwent repeat testing 6 months later allowing for n=229 total observations analyzed.

^a^Multiplicative interaction model was adjusted for race/ethnicity, sex, age, asthma diagnosis, obesity, heating season, DNA methylation on Day 1, personal BC on Day 1 and seroatopy or CR sensitization

^b^CpG position relative to the transcriptional start site

^c^Total IgE≥ 80 IU/mL

^d^Cockroach IgE ≥ 0.35 IU/mL

^e^Average methylation of ARG2 at CpG sites of -32, -30, and -26

**Table S5.** Effects of DNA methylation on FeNO measured 5 days later

| Gene | CpG promoter region^a^ | Beta coefficient^b^ [95% CI] |
| --- | --- | --- |
| IL4 | -326 | 0.93 [-1.86, 3.72] |
|  | -48 | **-1.06 [-1.83, -0.28]**** |
| IFNγ | -186 | -0.12 [-1.62, 1.39] |
|  | -54 | -0.21 [-0.85, 0.43] |
| NOS2A | +5099 | **-1.24 [-2.22, -0.22]*** |
|  | +5106 | -0.01 [-1.97, 1.95] |
| ARG2 | -32, -30, and -26^c^ | 0.03 [-0.07, 0.12] |

Note: 57 of the 127 children underwent repeat testing 6 months later allowing for n=184 total observations analyzed.

^a^CpG position relative to the transcriptional start site

^b^Adjusted for race/ethnicity, sex, age, asthma diagnosis, obesity, seroatopy, heating season, and ambient NO on Day 6

^c^Average methylation of ARG2 CpG sites of -32, -30, and -26

*p<0.05, and **p<0.01.

**Table S6**. Personal BC is associated with changes in DNA methylation (BDNA_2_-BDNA_1_) of IL4 CpG^-48^

| Gene | CpG promoter region^a^ | Beta coefficient^b^ [95% CI] |
| --- | --- | --- |
| IL4 | -326 | 0.13 [-0.38, 0.65] |
|  | -48 | **-1.75 [-3.48, -0.02]*** |
| IFNγ | -186 | 0.64 [-0.38, 1.67] |
|  | -54 | 0.87 [-0.22, 1.96] |
| NOS2A | +5099 | -0.59 [-1.82, 0.63] |
|  | +5106 | 0.91 [-0.07, 1.89] |
| ARG2 | -32, -30, and -26^c^ | 0.09 [-0.09, 0.26] |

Note: 86 of the 143 children underwent repeat testing 6 months later allowing for n=229 total observations analyzed.

^a^CpG position relative to the transcriptional start site

^b^Adjusted for race/ethnicity, sex, age, asthma diagnosis, obesity, seroatopy, and heating season

^c^Average methylation of ARG2 CpG sites of -32, -30, and -26

*p<0.05

**Pyrosequencing** Internal checks in quality assessment were those described by the manufacturer (Qiagen). All of the data analyzed “passed” all of these internal checks for: 1. High pre-sequencing, 2. Low signal-to-noise ratio, 3. Wide peaks, and 4. Possible dispensation error. The stringency level for CpG pattern deviation and CpG sum deviation were both set at “Normal,” and the allowed background signal for unsuccessful bisulfite treatment (checks included in each assay) was set at 4.5%.

**Pulmonary function tests (PFTs)** PFTs were conducted during in-home visits on Day 1 and Day 6 (Figure S1) using a portable spirometer (Koko, nSpire Health, Longmont, Colorado), in accordance with ATS and ERS guidelines [[33](#_ENREF_33)] and repeated 6 months later. Tests were considered acceptable if they met the following criteria: 1) rapid upstroke, 2) volume extrapolated <5% of FVC, 3) minimal premature termination of exhalation (premature termination=termination at >15% of peak flow), and 4) smooth exhalatory limb [[38](#_ENREF_38)]. PFTs that did not meet the acceptability criteria were excluded (n=14). Five spirometry outcome measures were included for analysis: FVC, forced expiratory volume in one second (FEV_1_), FEV_1_/FVC, forced expiratory flow at 25–75% of forced vital capacity (FEF_25-75_) and peak expiratory flow rate (PEFR).

**Statistical Analysis** Multivariable linear regression analyses were used to examine the five-day lag effects of DNA methylation (BDNA_1_) on each lung function outcome on Day 6, after controlling for race/ethnicity, sex, age, height, asthma diagnosis, obesity, seroatopy, heating season, and each lung function outcome measured on Day 1.

**Figure S1.** Conserved promoter regions


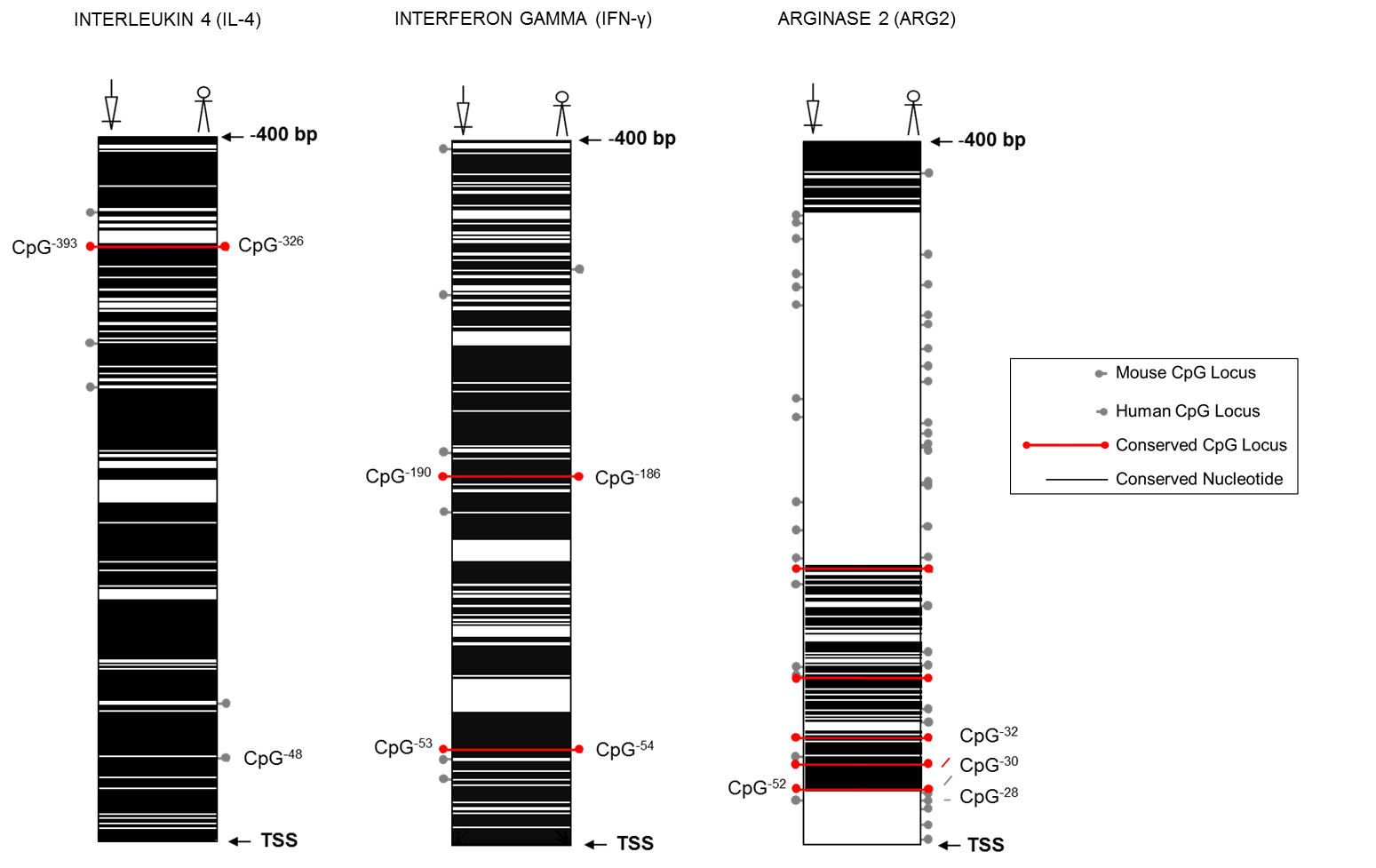


Arginase2 (ARG2)

Interferon gamma (IFNγ)

Interleukin 4 (IL4)

**Figure S2.** Schematic demonstration of collected measures.

**Figure S3.** Correlations between Day 1 and Day 6 buccal cell DNA methylations of a) IL4 (CpG^-326^,CpG^-48^), b) IFNγ (CpG^-186^,CpG^-54^), c) NOS2A (CpG^+5099^, CpG^+5106^), and ARG2 (average methylation of CpG^-32^, CpG^-30^, and CpG^-26^)
